# P-1124. Infectious Complications in Minimally Invasive Spine Surgery Over the Last Decade: A Systematic Review and Meta-Analysis

**DOI:** 10.1093/ofid/ofaf695.1319

**Published:** 2026-01-11

**Authors:** Mario Benvenutti-Regato, Misael Salazar-Alejo, Ana Ballesteros-Suarez, Ryan Luna-Fernandez, Rogelio Flores-Salcido, David Karam-Almada, Gabriela Guemes-Aguilar, Luisa C Cuellar-Araiza, Jose A Figueroa-Sanchez

**Affiliations:** Instituto de Neurología y Neurocirugía, Hospital Zambrano Hellion TecSalud, Monterrey, Nuevo Leon, Mexico; Tecnologico de Monterrey, Monterrey, Nuevo Leon, Mexico; Departamento de Medicina, Tecnológico de Monterrey, Escuela de Medicina y Ciencias de la Salud, Monterrey, México, Monterrey, Nuevo Leon, Mexico; Tecnologico de Monterrey, Monterrey, Nuevo Leon, Mexico; Universidad de Monterrey, Monterrey, Nuevo Leon, Mexico; Universidad de Monterrey, Monterrey, Nuevo Leon, Mexico; Tecnologico de Monterrey, Monterrey, Nuevo Leon, Mexico; Tecnologico de Monterrey, Monterrey, Nuevo Leon, Mexico; Instituto de Neurología y Neurocirugía, Hospital Zambrano Hellion TecSalud, Monterrey, Nuevo Leon, Mexico

## Abstract

**Background:**

Postoperative spinal infections are serious complications that can greatly affect patient outcomes after spine surgery, leading to both immediate risks and long-term consequences. Understanding their incidence is essential for enhancing prevention, early detection, and treatment strategies. This meta-analysis aims to assess the incidence of infectious complications in patients who underwent minimally invasive spine surgery for degenerative spine diseases over the past decade.

**Figure:**
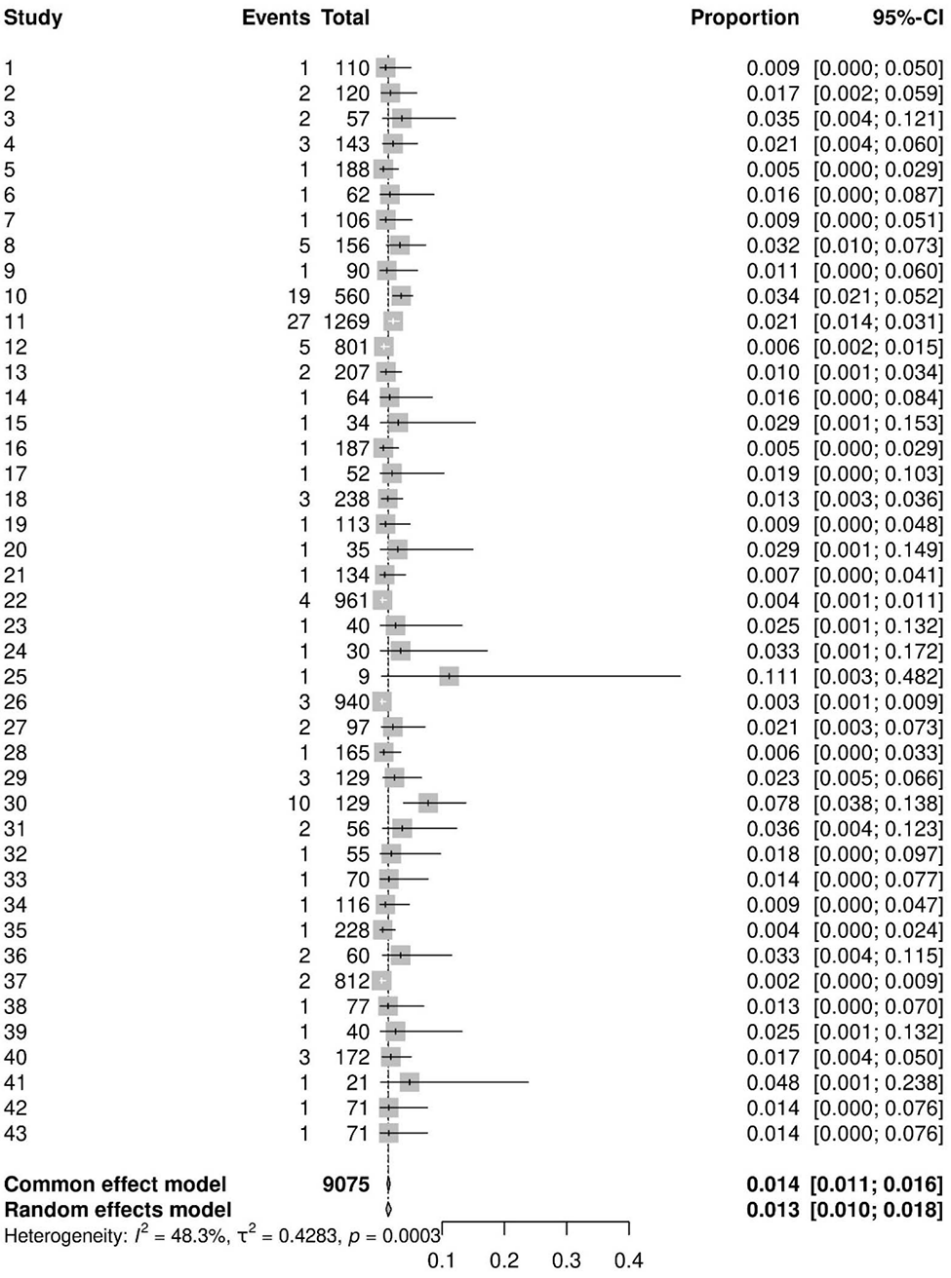
Superficial Skin Infection Incidence

**Figure:**
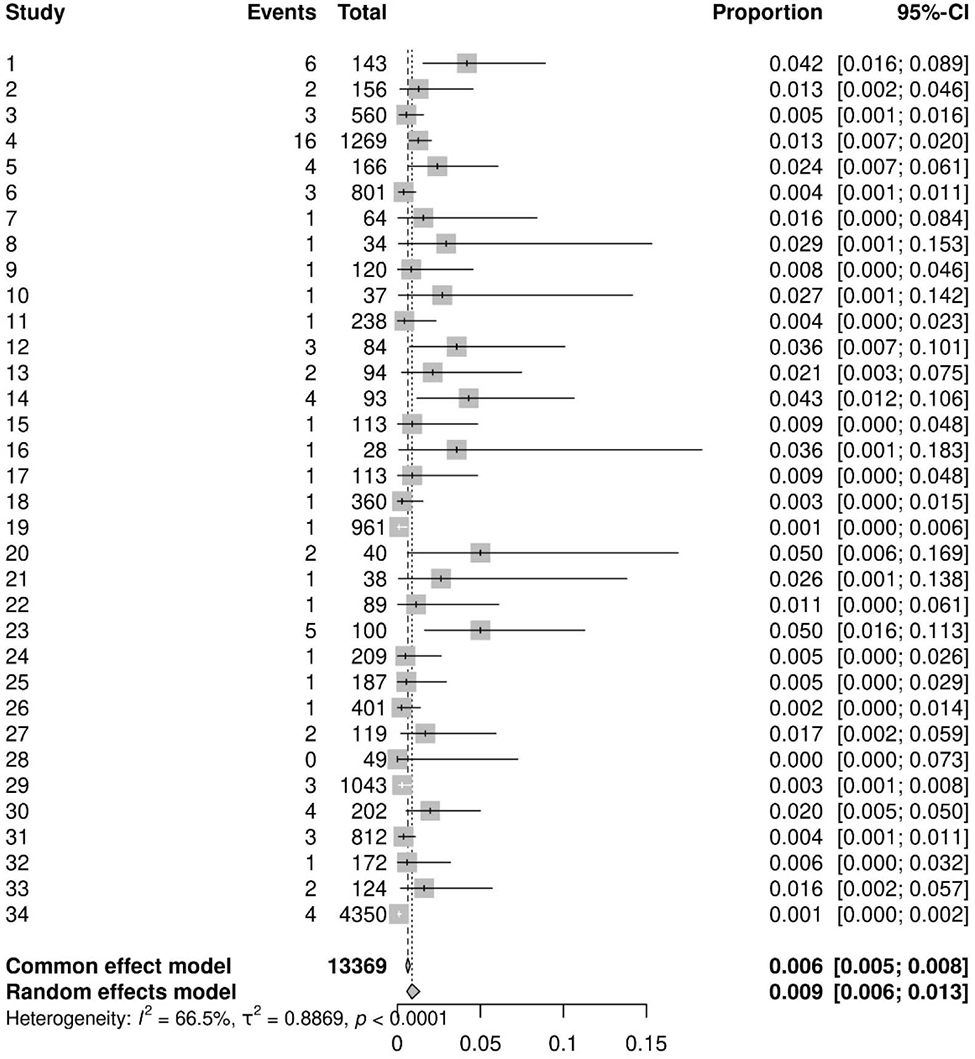
Deep Skin Infection Incidence

**Methods:**

A comprehensive search strategy was developed to query the MEDLINE, Scopus, and Web of Science databases for studies published in the past decade. After removing duplicate records, two independent reviewers assessed the remaining studies for eligibility. Primary studies that examined minimally invasive surgical techniques for treating degenerative spine diseases and provided complete data on the incidence of infectious complications were included. All infectious complications were considered, including superficial and deep surgical site infections, urinary tract infections, and pneumonia. Statistical analyses were conducted using generalized linear mixed models in R.

**Figure:**
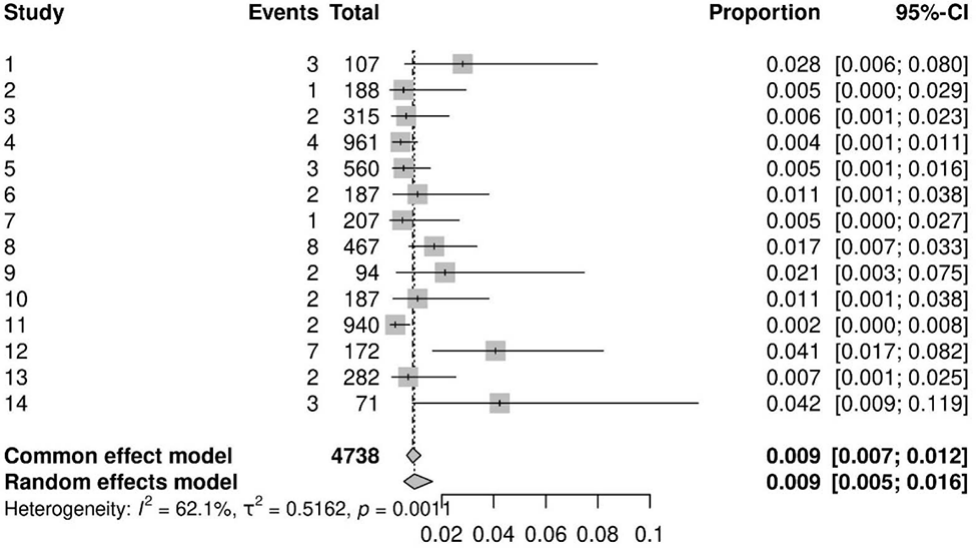
Pneumonia Incidence

**Figure:**
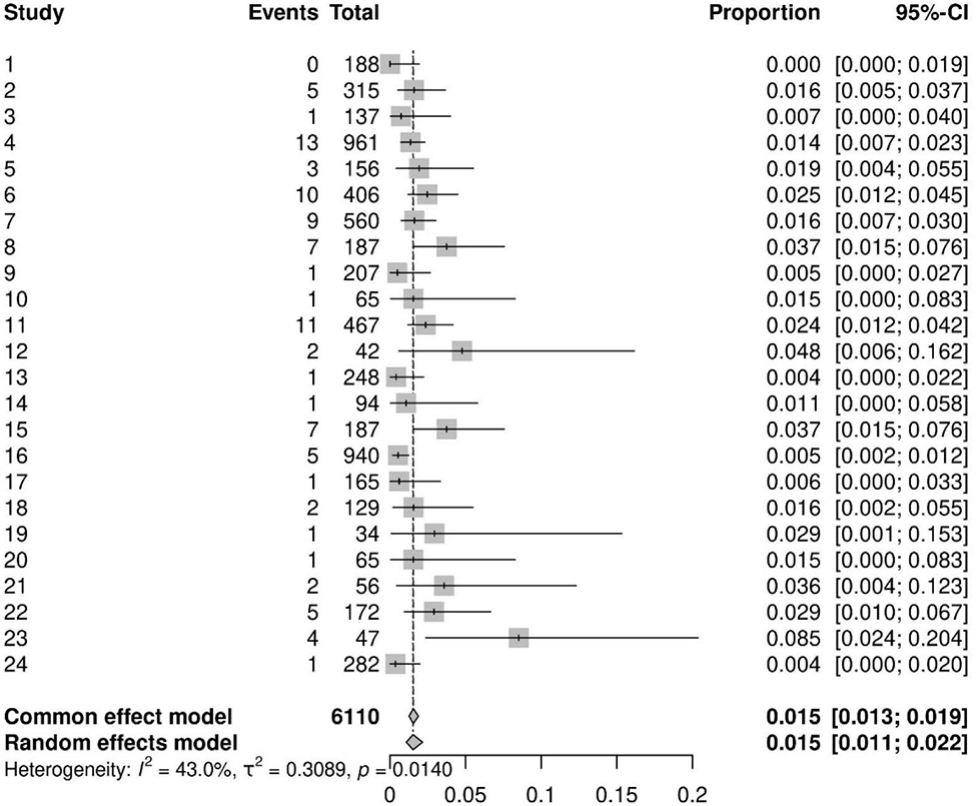
Urinary Tract Infection Incidence

**Results:**

A total of 219 studies, encompassing 36,285 patients, were analyzed. The majority (76%) were retrospective studies. The pooled incidence of superficial surgical site infections was 1.3% (95% CI, 1.0–1.8%), while deep surgical site infections occurred in 0.9% of patients (95% CI, 0.6–1.3%). Postoperative pneumonia was estimated to affect 0.95% of patients (95% CI, 0.54–1.65%), and urinary tract infections were observed in 1.5% of cases (95% CI, 1.1–2.2%).

**Conclusion:**

Our results indicate that postoperative infections following minimally invasive spine surgery may be relatively infrequent. However, several factors limit the reliability of this estimate, including the predominance of retrospective studies, a shortage of data from regions outside Asia, and inconsistent reporting of infectious complications. Further research employing standardized infection reporting criteria and prospective study designs is necessary to improve the accuracy and reliability of infection incidence estimates in minimally invasive spine surgery.

**Disclosures:**

All Authors: No reported disclosures

